# Supplementary figures and images for: Two distinct regulatory systems control pulcherrimin biosynthesis in Bacillus subtilis
Source: PLoS Genet. 2024 May 16;20(5):e1011283. doi: 10.1371/journal.pgen.1011283 (PMC11135676; doi:10.1371/journal.pgen.1011283)

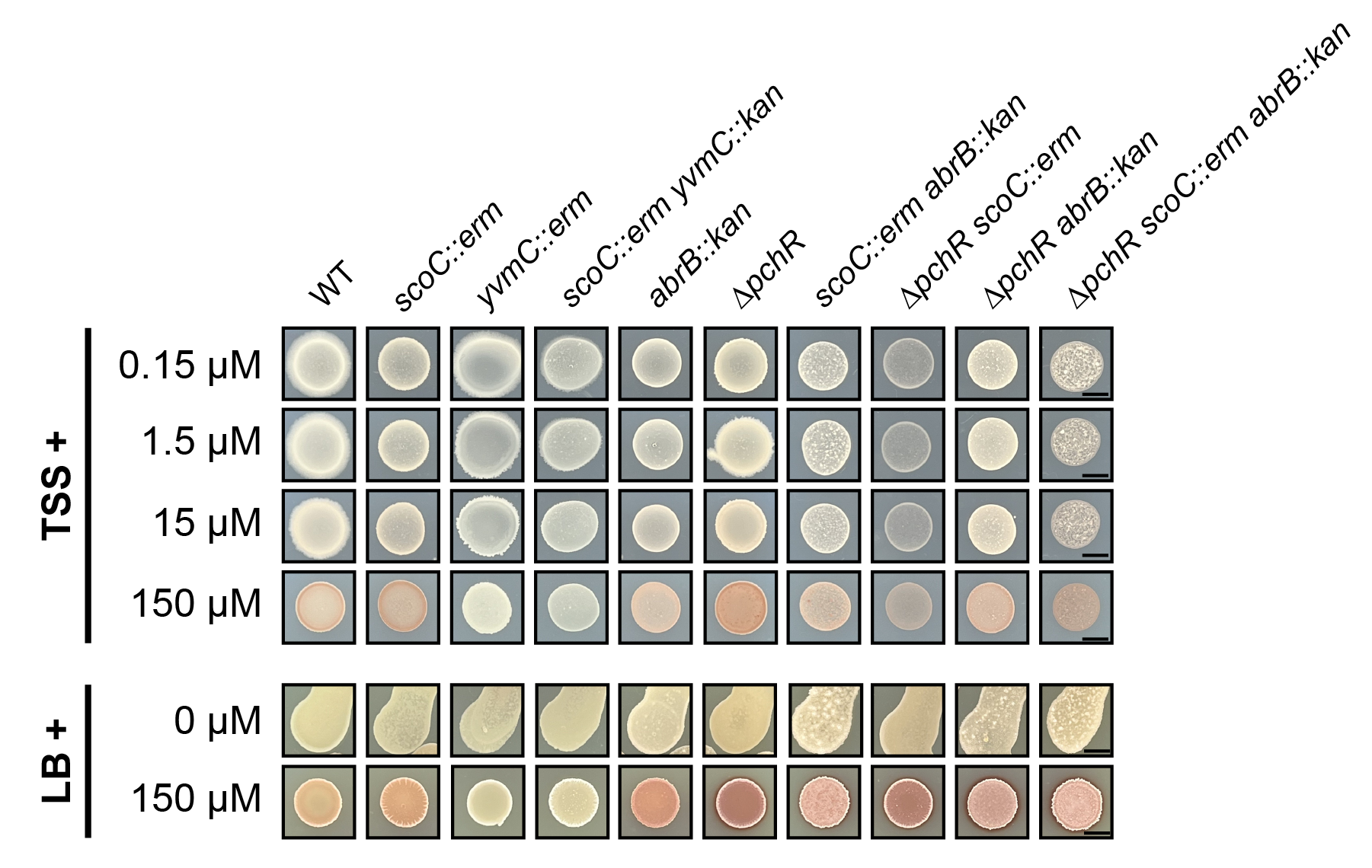

Supplement: S1 Fig — 10 μL spots of WT and isogenic mutants on TSS (top) or LB (bottom) supplemented with different concentrations of ferric citrate. The black scale bar represents 5 mm. (TIF) [file pgen.1011283.s001.tif]

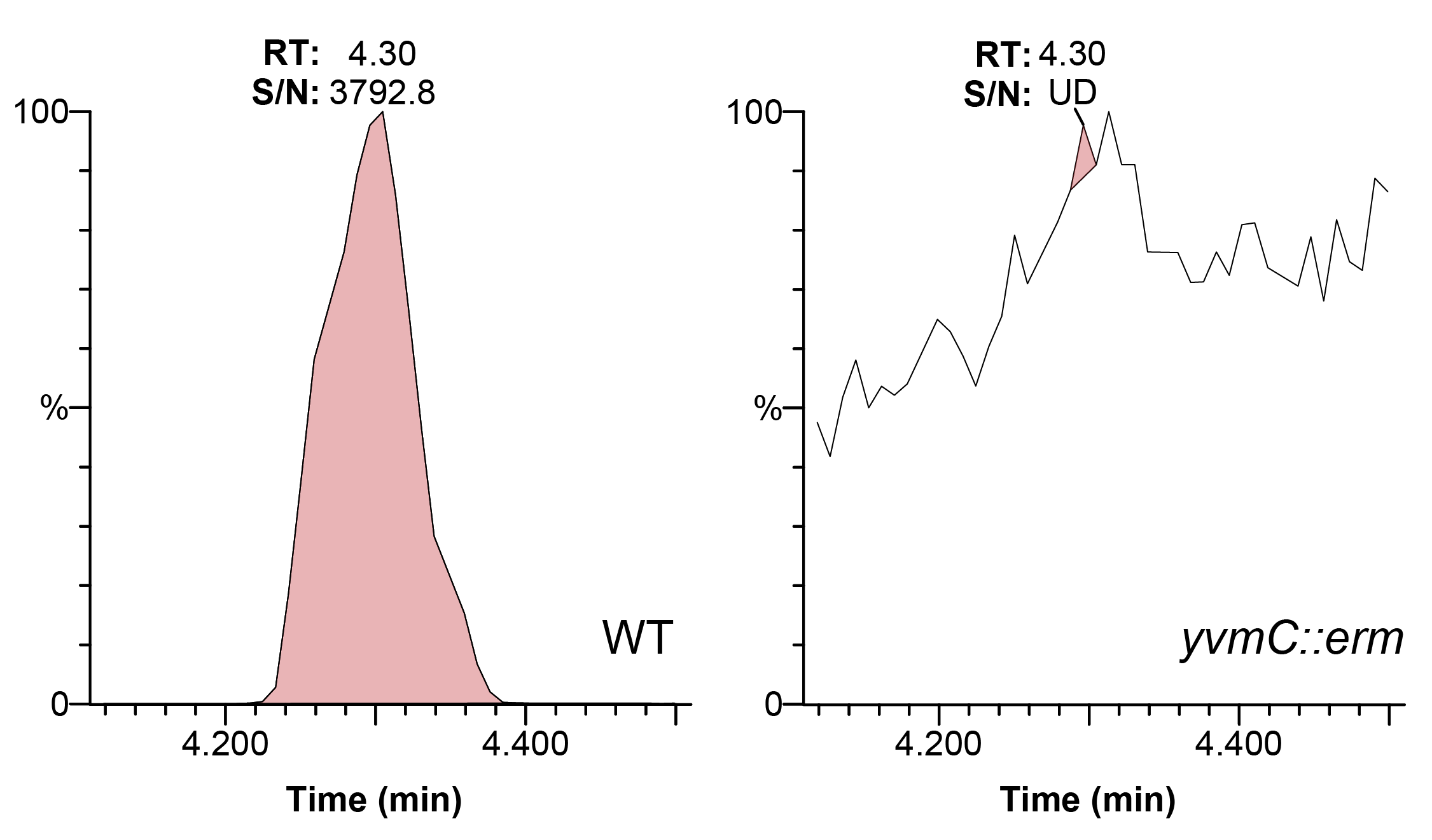

Supplement: S2 Fig — Metabolites were extracted from WT (A) and yvmC::erm (B) grown in liquid culture and were subject to mass spectrometry analysis for cyclo-(l-leucine-l-leucine), a precursor metabolite for pulcherrimin. The experiment was repeated at least three times with representative data shown. RT (retention time) and S/N (signal to noise ratio) for the peak corresponding to cLL are shown in each panel. The S/N ratio for yvmC::erm was under the limit for detection (UD, undetermined). (TIF) [file pgen.1011283.s002.tif]

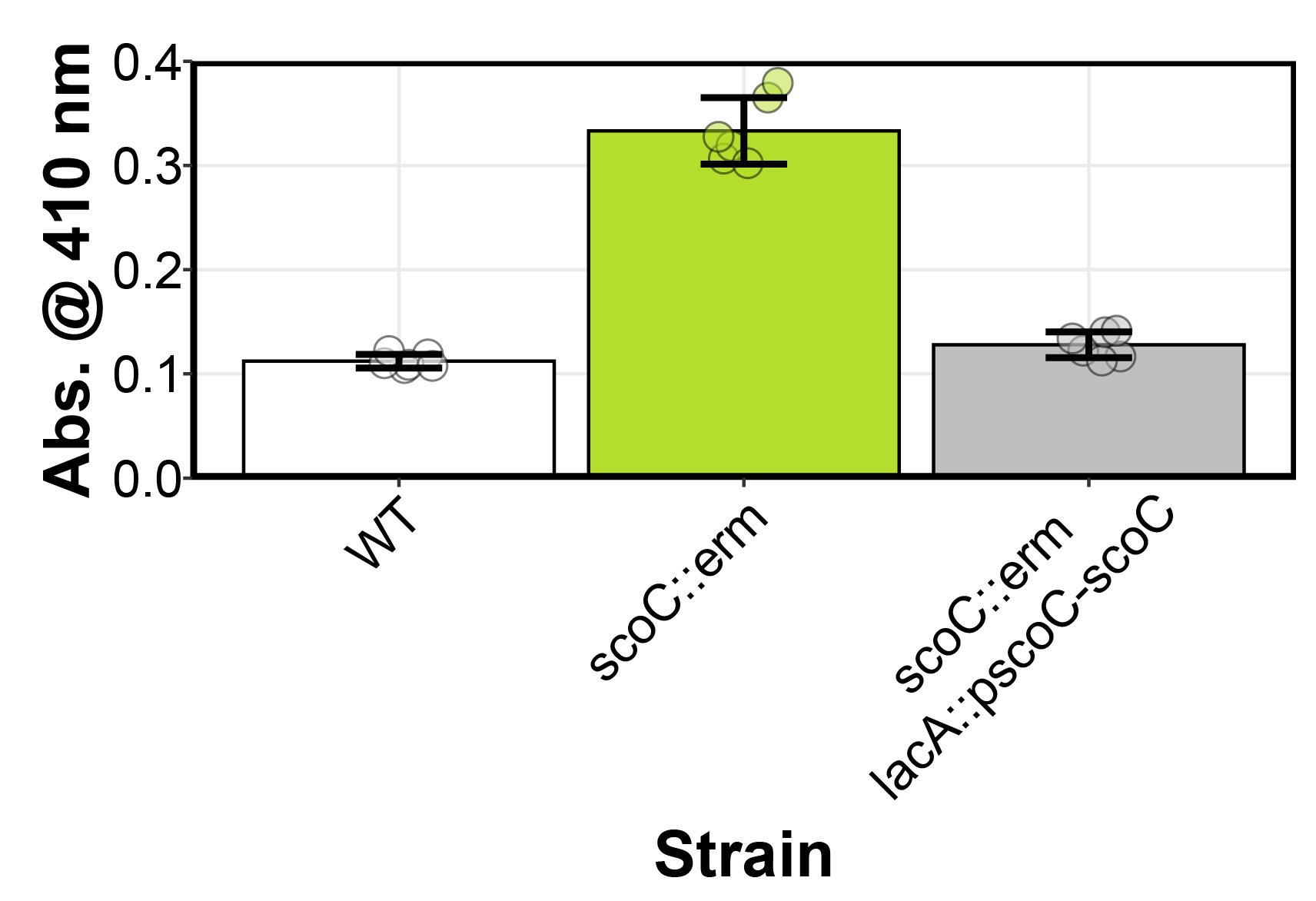

Supplement: S3 Fig — Liquid pulcherrimin measurements from WT, scoC::erm, and scoC::erm lacA::pscoC-scoC from late stationary phase cultures grown in TSS medium. Bars represent the mean A410 from five independent replicates. (TIF) [file pgen.1011283.s003.tif]

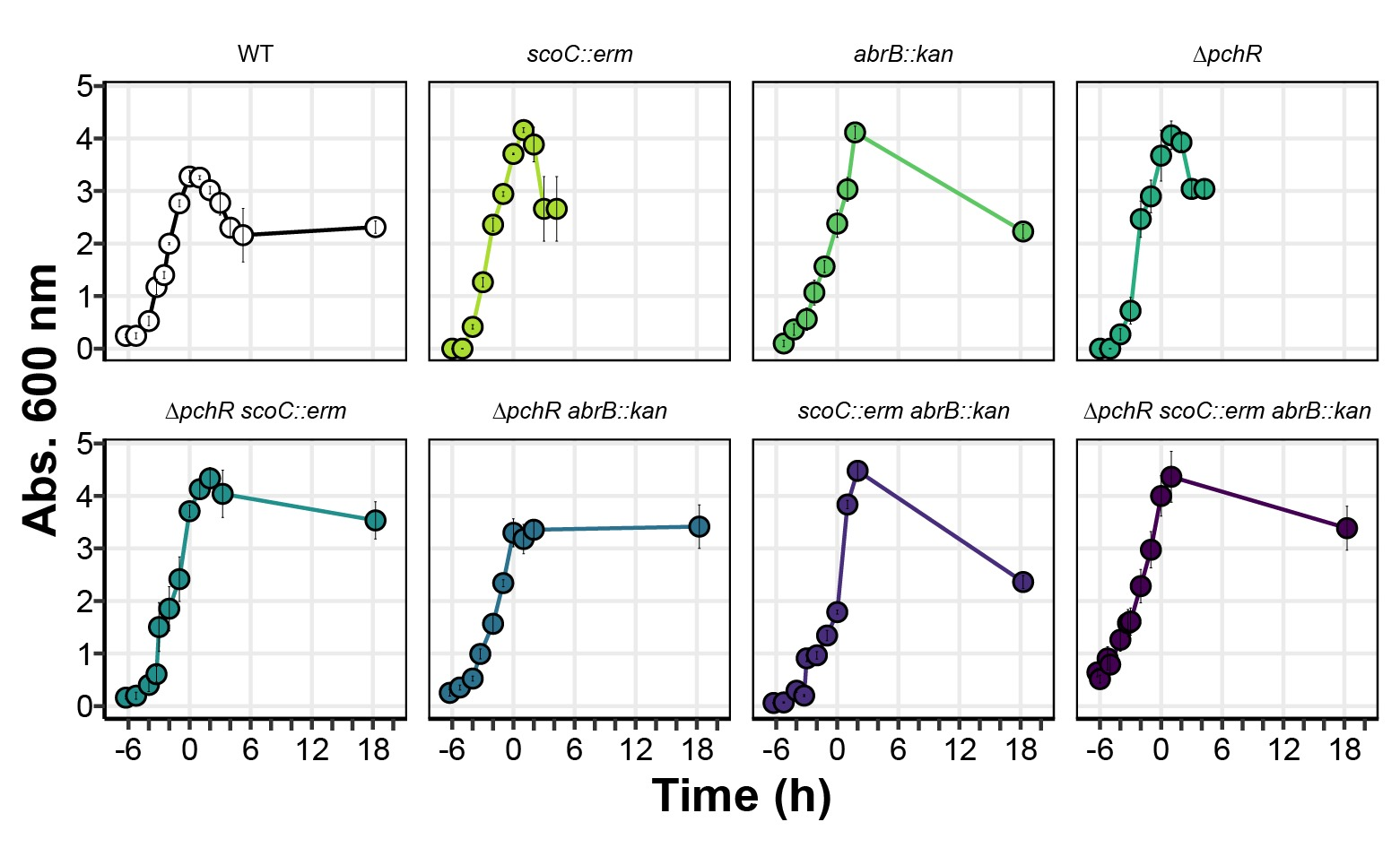

Supplement: S4 Fig — Circles represent average Abs. 600 nm and error bars represent standard deviation. (TIF) [file pgen.1011283.s004.tif]

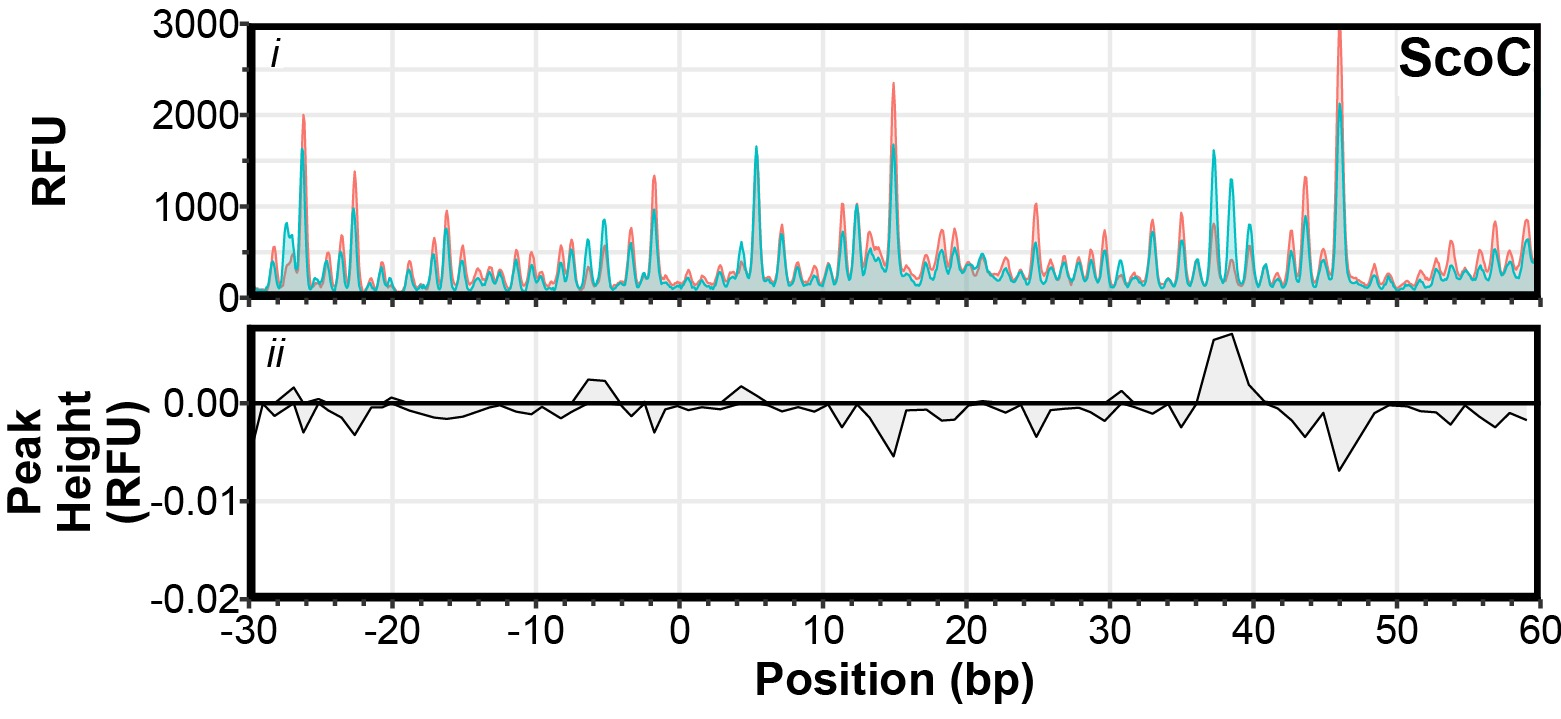

Supplement: S5 Fig — Similar to Fig 5C except heparin was added to the reactions. Fluorescent DNase I footprinting (i) and DFACE analysis with ScoC (ii). In the top panel, red electropherograms represent no protein while blue electropherograms represent reactions with protein. (TIF) [file pgen.1011283.s005.tif]
