# Supplementary material for: Two distinct regulatory systems control pulcherrimin biosynthesis in Bacillus subtilis
Source: PLoS Genet. 2024 May 16;20(5):e1011283. doi: 10.1371/journal.pgen.1011283 (PMC11135676; doi:10.1371/journal.pgen.1011283)
Supplement: S1 Table — (DOCX) [file pgen.1011283.s006.docx]

| **Strain** | **Genotype** | **Selection** |
| --- | --- | --- |
| DK1042 | *B. subtilis* NCBI 3610 ComI^Q12L^ | - |
| NF057 | *scoC::erm* | MLS |
| NF062 | *yvmC::kan* | Kan |
| NF064 | *scoC::erm abrB::kan* | MLS Kan |
| NF077 | *abrB::kan* | Kan |
| NF079 | *∆pchR abrB::kan* | Kan |
| NF081 | *∆pchR* | - |
| NF088 | ∆pchR scoC::erm | MLS |
| NF089 | *scoC::erm yvmC::kan* | MLS Kan |
| NF092 | *∆pchR scoC::cam abrB::erm* | Cam Erm |
| NF131 | *abrB::spec yvmC::erm* | Spec Erm |
| NF093 | *scoC::erm lacA::pscoC-scoC* | Cam Erm |
| NF102 | WT *amyE::pYvmC-GFP* | Cam |
| NF103 | *scoC::erm amyE::pYvmC-GFP* | Cam Erm |
| NF104 | *∆pchR amyE::pYvmC-GFP* | Cam |
| NF105 | *∆pchR scoC::erm amyE::pYvmC-GFP* | Cam Erm |
| NF117 | *abrB::kan amyE::pYvmC-GFP* | Kan Cam |
| NF106 | *∆pchR abrB::kan amyE::pYvmC-GFP* | Kan Cam |
| NF107 | *∆pchR ∆scoC abrB::erm amyE::pYvmC-GFP* | Erm Cam |
| NF118 | *scoC::erm abrB::spec amyE::pYvmC-GFP* | Erm Spec Cam |
