# Supplementary material for: Two distinct regulatory systems control pulcherrimin biosynthesis in Bacillus subtilis
Source: PLoS Genet. 2024 May 16;20(5):e1011283. doi: 10.1371/journal.pgen.1011283 (PMC11135676; doi:10.1371/journal.pgen.1011283)
Supplement: S2 Table — (DOCX) [file pgen.1011283.s007.docx]

| **Name** | **Description** | **Resistance** |
| --- | --- | --- |
| fNLF001 | abrB::erm with flanking homologous regions | Erm |
| fNLF002 | abrB::kan with flanking homologous regions | Kan |
| fNLF017 | abrB::spec with flanking homologous regions | Spec |
| fNLF003 | pchR::erm with flanking homologous regions | Erm |
| fNLF005 | yvmC::kan with flanking homologous regions | Kan |
| fNLF011 | yvmC::erm with flanking homologous regions | Erm |
| fNLF015 | scoC::camR-pscoC-FLAG-ScoC with flanking homologous regions | Cam |
| fNLF016 | scoC::camR with flanking homologous regions | Cam |
| pNF035 | pYvmC in pLVG001 | Spec |
| pNF038 | lacA::pscoC-ScoC | Cam |
| pNF039 | PchR in pE-SUMO | Kan |
| pNF040 | AbrB in pE-SUMO | Kan |
| pTMN007 | ScoC in pE-SUMO | Kan |
| pNF047 | pYvmC in pGFP-Star | Cam |
