# Supplementary material for: Two distinct regulatory systems control pulcherrimin biosynthesis in Bacillus subtilis
Source: PLoS Genet. 2024 May 16;20(5):e1011283. doi: 10.1371/journal.pgen.1011283 (PMC11135676; doi:10.1371/journal.pgen.1011283)
Supplement: S3 Table — (DOCX) [file pgen.1011283.s008.docx]

| **Name** | **Primer Description** | **Sequence (5′-3′)** | **Amplicon Description** |
| --- | --- | --- | --- |
| oNLF336 | abrB_KO_US_For | atagtatttcagaagacgatccgc | AbrB::AbR disruption |
| oNLF337 | abrB_KO_US_Rev | ctctcctttctcgcctgccattctcctcccaagagata |  |
| oNLF338 | abrB_KO_DS_For | gcagtgacaggagcctcgtaatcatttcttgtacaaaa |  |
| oNLF339 | abrB_KO_DS_Rev | aatgtaaggacaatagctggtatgc |  |
| oNLF581 | abrB_Spec_US_Rev | tttgttcgtatgtattcaaatatatcctcccattctcctcccaagagatac |  |
| oNLF584 | abrB_Spec_DS_For | aactatttaaataacagattaaaaaaattataatcatttcttgtacaaaaaacg |  |
| oNLF340 | pchR_KO_US_For | tactgatcttctcacccagcttc | PchR::AbR disruption |
| oNLF341 | pchR_KO_US_Rev | ctctcctttctcgcctgccataggctaccttctttctt |  |
| oNLF342 | pchR_KO_DS_For | gcagtgacaggagcctcgtaaacaaaaaggcggtgtac |  |
| oNLF343 | pchR_KO_DS_Rev | cattgtgagcaagtaggcagatac |  |
| oNLF344 | yvmC_KO_US_For | tcggtttgttctgcttcaagt | YvmC::AbR disruption |
| oNLF345 | yvmC_KO_US_Rev | ctctcctttctcgcctgccatctcattcacccctaaaa |  |
| oNLF346 | yvmC_KO_DS_For | gcagtgacaggagcctcgtgatagggggagtaaaacat |  |
| oNLF347 | yvmC_KO_DS_Rev | gtcaggctgttcaaatgcttc |  |
| oNLF356 | AbR_KO_For | gcaggcgagaaaggagag | AbR Amplification |
| oNLF357 | AbR_KO_Rev | cgaggctcctgtcactgc |  |
| oNLF432 | pLVG001_For_FAM | gctgcaggaattcgactctc | 5' FAM and 5' IRD700 probe (WT and ∆59) for DNAse I footprinting and EMSA |
| oNLF433 | pLVG001_For_IRD700 | gctgcaggaattcgactctc |  |
| oNLF387 | yvmC_promoter_reverse_pDR110 | catgtttgtcctccttattagttaatcagctagctccggtcatctcattcacc |  |
| oLVG025A | Amplifies pDR110 backbone, with oNLF467 | ctcttgccagtcacgttacg |  |
| oNLF467 | yvmC_scoC_delta_us_rev | taataatcattttcaccaaacgtcaatatgatctgtg |  |
| oNLF468 | yvmC_scoC_delta_ds_for | catattgacgtttggtgaaaatgattattaaaatcttaaaaaacatttg |  |
| oNLF407 | scoC_us_fwd | ctatcgtcagctttattgatc | scoC::cam disruption |
| oNLF408 | scoC_us_rev | cgctctcctttctcgcctgccattacgtcacctgcttc |  |
| oNLF409 | camR_fwd | gcaggcgagaaaggagagcgtcaggtggcacttttcg |  |
| oNLF410 | camR_rev | cgaggctcctgtcactgcgacattagaaaaccgactgtaaaaag |  |
| oNLF411 | scoC_ds_fwd | tcgcagtgacaggagcctcgaagagctcgaacctgtaaac |  |
| oNLF412 | scoC_ds_rev | aacaagaatatccaagccg |  |
| oNLF471 | lacA_US_For_pBR332 | gtaatgataccgatgaaacgagaggcgggacagatatcctcg | Native ScoC complementation at *lacA* |
| oNLF472 | lacA_US_Rev_pBR332 | gtgccacctgactcacattctcctccttgttc |  |
| oNLF473 | CamR_for_lacA | aggagaatgtgagtcaggtggcacttttcg |  |
| oNLF474 | pscoC-ScoC_rev_lacA | cggagcatcagcttaactgtttacaggttcg |  |
| oNLF475 | lacA_ds_for_pBR332 | gtaaacagttaagctgatgctccgctcgatatg |  |
| oNLF476 | lacA_ds_rev_pBR332 | acctacatctgtattaacgaagcggcctccattacatctcttactgc |  |
| oNLF495 | abrB_pET-SUMO_for | attgaggctcaccgcgaacagattggaggtatgaaatctactggtattgtac | AbrB Purification Vector |
| oNLF496 | abrB_pET-SUMO_rev | gatctcagtggtggtggtggtggtgctcgattatttaaggttttgaagctg |  |
| oNLF497 | pchR_pET-SUMO_for | attgaggctcaccgcgaacagattggaggtatgtctgatttgacaaaacag | PchR Purification Vector |
| oNLF498 | pchR_pET-SUMO_rev | gatctcagtggtggtggtggtggtgctcgattactttacaggtttgtctg |  |
| oNLF524 | yvmC_upstream_for | ttacagtcattttaccgcgggctttcccatgtgatgtttacatttattttcaaatatttg | pyvmC-GFP integration vector |
| oNLF525 | yvmC_upstream_rev | cgttaccattccggtcttatcccgctttaagtc |  |
| oNLF526 | yvmC_downstream_for | ttaaagcgggataagaccggaatggtaacggaaag |  |
| oNLF527 | yvmC_downstream_rev | ctattgaatccatagtagttcctccttccctaaattgagctttcgccc |  |
