# Supplementary material for: Two distinct regulatory systems control pulcherrimin biosynthesis in Bacillus subtilis
Source: PLoS Genet. 2024 May 16;20(5):e1011283. doi: 10.1371/journal.pgen.1011283 (PMC11135676; doi:10.1371/journal.pgen.1011283)
Supplement: Attachment — Submitted filename: Reviewer_Responses_Fernandez.docx [file pgen.1011283.s010.docx]

We would like to begin by thanking the reviewers for their insightful comments that have improved the quality and clarity of our work.

# Review 1

## Summary:

The authors characterize the roles of two proteins ScoC and AbrB in the repression of pulcherrimin synthesis in B. subtilis. Pulcherrimin is a somewhat unusual compound that sequesters iron extracellularly and is made when iron levels are high, rather than low. They serendipitously notice that a strain lacking the DNA binding protein ScoC overproduces pulcherrimin in liquid culture. They also show that AbrB also represses pulcherrimin genes, as was predicted by bioinformatics analysis but not previously shown. Finally, they make a strain lacking ScoC, AbrB and PhcR and it additively hyperproduces pulcherrimin with the largest contribution made to repression by PhcR. Biochemically, all three proteins bound to the PyvcM promoter, and while AbrB and ScoC binding was much weaker, I think all three proteins bound to the same 59 base pair region within the promoter. All told, the figures and physiological data are extremely nice. The biochemistry I was less confident in, particularly the footprinting as AbrB and ScoC protection seemed rather nonspecific. Finally, I think these results will help us get a better understanding of what pulcherrimin is, what it does, and when/where it does it, but I find myself hard pressed to articulate specifically what it tells us. Finally, the model is appropriately vague, but it feels uncomfortable to have three proteins binding to the same place and have additive effects rather than stepwise effects dominated by proteins in order of highest affinity.

## We appreciate the reviewer’s positive comments on our work. We have also revised the manuscript to more clearly articulate the importance of our work to the field including the addition of a new model in Fig 7.

## Specific Points

1) Line 55 and title. Why do these lines say two distinct regulatory systems when three different regulators are discussed (PhcR, ScoC, AbrB)?

We thank reviewer #1 for their comments. We chose the wording “two distinct regulatory systems” rather than “three different regulators” because we wanted to focus more on the regulatory networks the transcription factors belong to: 1) transition state regulators (AbrB and ScoC) and 2) pulcherrimin specific regulation (PchR).

2) Line 106. Clarify “under control”. Are the genes activated or repressed?

We have rewritten this section of the introduction and have clarified that the genes are negatively regulated by PchR.

3) Line 467. “positive epistasis”. Do you mean an “additive effect” or “synergy”? Epistasis typically refers to a double mutant having the phenotype of one of the single mutants.

The use of “positive epistasis” was used to specify which type of epistasis was occurring. In the text, we use epistasis to define phenotypic changes that would not be expected by the additive effects of combining mutations. Specifically, when the combined effects of two mutations is greater than the additive assumption, this situation is defined as *positive* epistasis (which would be synonymous with synergy in this context). While not observed in our study, if the combined effects of two mutants resulted in a phenotype *less than* the additive assumption, this result is referred to as *negative* epistasis. The use of positive epistasis in this context was informed by the following study, which has become a benchmark for terminology centered on epistasis analysis [1]**.**

4) Are the biomass yields the same for each mutant? Just curious as to whether pulcherimin production is dependent on the number of bacteria making it.

We thank the reviewer for this comment. We have included the OD_600_ averages for each strain taken during the experiment for newly updated Figure 3 with the OD600 data presented as new S4 Fig. From the growth curves for the double and triple mutants which produce the most pulcherrimin, the maximum OD_600_ values range between 3.5 to 4.5. Additionally, the mutants have similar growth trajectories throughout the transition from exponential to stationary phase. Thus, the differences in pulcherrimin production represent differences in the production rate of pulcherrimin rather than the total number of cells producing pulcherrimin. Analysis of the cellular growth curve has been included in the beginning of the results section for updated Figure 3.

5) Line 496. It is curious that the reporter would have a genetic effect. The following line suggests construction was attempted by transformation into a strain defective in transformation. Have you tried transduction? Or are the authors thinking it has something to do with either a cis or trans factor encoded within the promoter region of yvmC?

We have not tried transduction. We think it has something to do with the combination of antibiotic resistance markers, because we were able to make the strain when using a spectinomycin disruption of *abrB::spec* introduced into the *scoC::erm amyE::PyvmC-GFP cmR* background. With this new strain made, we were able to assess *PyvmC-GFP* activity and found that, like pulcherrimin production, expression of *PyvmC* was higher in the double mutant compared to the *scoC* and *abrB* mutant. We have updated now Figure 4 and the corresponding results section in the main text.

6) Line 522. Something about the term “mutant probe” really threw me off. Mutant usually refers to an in vivo alteration to the sequence. Here a region was deleted from an in vitro target. This may be an overreaction but I really got hung up here because I couldn’t understand how deleting the -10 box would be helpful for in vivo analysis. Mutant may be common usage in biochemistry I’m not sure. Maybe “mutated (or more specifically: deletion) probe”?

We appreciate the point made by the reviewer. We agree that deleting the -10 region would not provide information regarding in vivo analysis. In the context of the manuscript, mutant was used to differentiate between the WT probe. We can see how without further qualifiers, “mutant” alone could be ambiguous. Therefore, we have changed “mutant” or “mutant probe” when discussing the in vitro generated deletion to “∆59 probe” to more accurately reflect the changes made relative to the full-length probe.

7) Paragraph starting line 521. This paragraph is very long, and covers three different proteins. It could possibly be broken into setup and PhcR (known), with a separate paragraph(s) dedicated to the two new candidates. Or split into EMSA and footprint paragraphs. Whatever the case, it read as too dense and difficult to follow especially for the final section of results where a strong point is needed.

We appreciate the comment by the reviewer. We have divided the last paragraph into multiple paragraphs delineated by protein (PchR, AbrB, and ScoC) and experiment type (Footprinting and then EMSA).

8) Is it correct that DNA binding of PhcR, AbrB and ScoC were all abolished when using the D59 deletion?

Yes, it is correct that DNA binding of PchR and AbrB was abolished when using the ∆59 promoter construct. While there is still shifting at the highest ScoC concentration for the ∆59 promoter, we interpret this as non-specific binding caused by the high concentration of ScoC in the *in vitro* assay.

9) Line 554. Which seemingly redundant pathways are being referred to here? Clarify.

After further consideration, this statement is vague and unnecessary. Therefore, we’ve removed it from the Discussion section and left the remaining paragraph as is. We thank the reviewer for pointing this out.

10) Line 557. Does the triple mutant that overexpressed pulcherrimin have a growth defect, or suffer numerically in competition when grown with the wild type?

We appreciate this comment from the reviewer. This was partially addressed with the response to comment #4. While we have not done a competition experiment, we have included the growth curves for all strains in Figure 3 **(S4 Fig.)**. Compared to the WT, the triple mutant had a higher maximum OD_600_ and appears to have a similar growth rate during exponential phase (comparing the slopes from -6 to 0 h). However, we do note that the triple mutant had an extended lag phase compared to other strains (~8-10 hours compared to 3-4 hours for all other strains). Thus, one would hypothesize that the triple mutant would suffer competitively if co-cultured with the WT background due to the extended lag phase.

11) Line 584. Clarify “transcriptionally regulated”. Are they activated or repressed?

We have changed “transcriptionally regulated” to “negatively regulated at the transcriptional level”.

12) Line 620. “No pigment was produced”. Implicit in this sentence and the next is that pigmentation (color) is the product of pulcherrimin conjugated to iron suggesting that the pulcherrimin was pre-existent at the time of iron addition so that color change was immediate. Do I have this right?

Correct – however, the same day that we submitted this manuscript, another article was published describing the allosteric regulation of the PchR homologue in *Bacillus licheniformis.* Considering this new information, we have changed the discussion section regarding PchR regulation in *B. subtilis* and have removed this section from the discussion.

13) Figure 1. Do the authors have either a double mutant of abrB ycmC or a quadruple mutant of pchR abrB scoC yvmC? This could control for other siderophores/pigments under AbrB control. I ask because the control was done for scoC but not abrB.

This is a very good point and we have generated an *abrB::spec yvmC::erm* double deletion background and included colony and culture phenotype in updated Figure 1. Similar to the *scoC::erm yvmC::kan* double deletion, the *abrB::spec yvmC::erm* double deletion appeared white on TSS agar and grey when grown in TSS media, similar to the *yvmC::erm* single deletion, indicating the red color in the *abrB::erm* background was caused by pulcherrimin production.

14) Figure 3A. The gray bars are a wild type lacking the GFP reporter. Are the colored bars only the intensities of the subpopulations that express the reporter? Could the no GFP control be included as a separate panel and then the histograms of both the “on” and “off” subpopulations be shown for the mutants? This way, the bias of expression could be visually represented as well as the intensity maxima. Or perhaps the whole population is shown and the percent off is being calculated as the part that overlaps with the control? Whatever the case, please describe how the percent expressed is being determined.

We thank the reviewer for this comment. The colored bars represent the whole population. The percent off is calculated by taking the percent of events above the maximum from the no GFP control. The Methods section was expanded to better describe which values are included in Figure 3AB and how percent GFP positive was calculated.

# Reviewer #2

## Summary

The biofilm-forming and plant-roots associated Gram-positive soil bacterium Bacillus subtilis synthesizes (pulcherriminic acid) and excretes an iron chelating compound (pulcherrimin). It is thought that pulcherrimin serves as an agent that creates a zone of iron limitation around the cell/colony thereby hindering the growth of microorganisms competing for the same ecological niche. Other data associate pulcherrimin production with a role in increasing the resistance levels of the B. subtilis cells against toxic reactive oxygen species. – The genes encoding the enzymes for pulcherriminic acid synthesis cluster with a gene encoding a MarR-type regulator (PchR) and a gene for an export system (YvmA) for pulcherriminic acid (see Fig.6).

In this manuscript, N.L. Fernandez and L.A. Simmons have studied the transcriptional regulation of the operon encoding the pulcherriminic acid synthesis cluster and their data show that it is regulated in an intricate and intertwined fashion by two – more general acting - transition state regulators (AbrB; ScoC) and the more system-specific PchR repressor.

The data presented by the authors are novel and further substantially our understanding of the factors that control an ecophysiological important compound, pulcherrimin. While other studies have already addressed to some extend the genetic control mechanisms for production of pulcherriminic acid, the data presented in this manuscript go well beyond the already known facts. They also correlate the synthesis of pulcherriminic acid/ pulcherrimin with the growth phase of the cells. Hence, in this study genetic control is integrated with a view on physiological relevant parameters for growth (exponential phase versus stationary phase).

The manuscript is written in a very clear and concise fashion, thus aiding the readers in their understanding why a particular experiment was conducted and what the outcome of the experiment was. The experimental set-up of the study is well-controlled, and the description of the Methods section is excellent.

Consequently, I have no major criticism of the data or the interpretation presented in the manuscript but I have some suggestions that the authors might want to consider to improve the presentation of the manuscript.

We thank the reviewer for their positive comments about our work.

## Specific Points

1) While the authors clearly spell out the current concepts of the physiological roles of pulcherriminic acid/ pulcherrimin, they do this only in the “Discussion” (lines 627-631). I suggest that this important topic should be taken up already in the “Introduction” to the readers can more readily grasp the physiological context of the genetic studies conducted by the authors.

This is an important comment from the reviewer. We have included more background on the proposed physiological roles of pulcherrimin in the introduction.

2) In an unusual fashion, the authors refer to Fig. 6 in the “Introduction” (line 103) before they refer to Fig 1. The convention is that figures should be numbered according to the ranking they are referred to in the main text. – Having said that, I find Fig. 6 very important for aiding the reader to comprehend the genetics and physiology of the entire presented story. So, I am suggesting the Fig. 6A,B will become Fig 1. – The current Fig. 6B can also be improved somewhat by being more precise in indicating the positions of the DNA-segments recognized by PchR, ScoC and AbrB, as deduced by the DNA-foot-printing analysis conducted by the authors.

3) In this vain, it would be helpful that have a Fig (perhaps for the Suppl) that shows the actual DNA-sequence of the regulatory region (represented by the extended bar in Fig. 6B) and highlights the actual regions protected in the foot-printing analysis of the various tested transcription factors.

We thank the reviewer for their suggestions with Comments #2 and #3 and for the model figure. We have made the following changes to address the concerns: **1)** we moved the introductory model figure depicting synthesis, transport, and regulation with the relevant proteins to Figure 1 and **2)** we created a figure with two parts depicting the locations of the protected regions along with other relevant features of the *yvmC* promoter and a schematic model of pulcherrimin regulation by PchR, ScoC, and AbrB. We believe these updates improve the readability and provide a more concise model of how pulcherrimin is regulated.

4) The authors have focused their analysis on the operon (yvmC-cypX) encoding the enzymes mediating pulcherriminic acid synthesis (this is fine!). However, one wonders what is known about the gene-cluster (operon?) encoding the pchR regulator and the YvmA pulcherriminic acid exporter. Is pchR autoregulated by its gene product as is observed for many other genes encoding MarR-type regulators)? Any information available for a potential regulatory role of AbrB and ScoC for the pchR-yvmA gene cluster). – This issue should be addressed in the Discussion, as one would expect that the transcription of the gene for the export system would be similarly regulated as those encoding the biosynthetic enzymes.

This is an interesting comment from the reviewer. Please see our responses below the individual points raised:

- *What is known about the gene-cluster (operon?) encoding the pchR regulator and the YvmA pulcherriminic acid exporter? Is pchR autoregulated by its gene product as is observed for many other genes encoding MarR-type regulators)?*
  - According to the online database Subtiwiki, it appears the tandem genes *pchR-yvmA* are co-transcribed from the same promoter and can be considered an operon [2]. Additionally, deletion of *pchR* causes an increase in *pchR* promoter activity and, thus, also results in an increase in *yvmA* transcription [3]. *In vitro* biochemical analysis of the *pchR* promoter also demonstrated that PchR can bind to its own promoter at PchR-box binding sites [3].
- *Is there a potential regulatory role of AbrB and ScoC for the pchR-yvmA gene cluster?*
  - Interestingly, an AbrB binding site was identified in the *pchR* promoter by Chumsakul *et al.* This site overlapped the core promoter region and was upstream of two tandem PchR-box motifs however no putative ScoC binding site was found [4,5]. This suggests *abrB* negatively regulates *pchR-yvmA* expression, yet, analysis of the *yvmA* and *pchR* transcripts only found a small (0.7 and 0.9 fold-decrease, respectively) in the *∆abrB* background compared to the WT [4,5]. Thus, with the given information, it does not appear AbrB and ScoC directly regulate the *pchR-yvmA* operon but needs to be confirmed experimentally.
- *One would expect that the transcription of the gene for the export system would be similarly regulated as those encoding the biosynthetic enzymes.*
  - In the mutants we examined, while not tested directly, there is evidence that pulcherrimin is exported into the supernatant: 1) when cells were collected by centrifugation, a brownish-yellow cell pellet is present with a red, pulcherrimin pellet present with a clear color demarcation between the two pellets, indicating the bulk of pulcherrimin is outside of the cells. Additionally, when spotted onto TSS plates or LB plates supplemented with iron, a red halo of pulcherrimin was observed around the colony after continued incubation (greater than 24 hours). Therefore, pulcherrimin is likely exported from the cells in all of the mutants tested. It is possible that the basal expression of *pchR-yvmA* provides sufficient amount of the transporter for the conditions tested.
- Also, in response to this important comment we have added a section regarding the potential regulation of *pchR-yvmA* and its implications in the discussion.

# Reviewer #3

# Summary

This is a solid and convincing study on the impact of 3 regulators, AbrB, ScoC, and PchR on the expression of genes responsible for pulcherimic acid biosynthetsis in B. subtilis. The finding that ScoC is involved is the only truly novel aspect of this regulation, since both PchR and AbrB were already known or indicated to be involved. Nevertheless, this thorough analysis provides a first comprehensive and convincing treatise on regulation of the chelator. But it remains are rather specific affair for a rather specific audience. The experiments are well-designed, the conclusions justified, the Figures suitable to get the major points across.

## Specific Points

1) Line 400: Since this Fig. is cited here for the first time, it should be Fig. 2, right?! Please change order of Figures accordingly.

The reviewer is correct – the model figure is now Figure 1 as it addressed first in the revised manuscript and the remaining figures have been updated.

2) Line 496: Any ideas as to why the double mutant, when combined with the reporter, cannot be generated? After all, the double mutant itself could be constructed.

We appreciate the comment from the reviewer. While we do not know exactly why we were unable to generate the double mutant in the background of the reporter, we suspect it was to do with the combination of resistance markers in the strain. As addressed in the response to comment #5 from reviewer 1, we had made this strain using a different antibiotic resistance marker (*scoC::erm abrB::spec amyE::PyvmC-GFP cmR*) and have updated Figure 3AB and the corresponding results section.

3) Line 511: This sentence should not be in bold face.

We thank the reviewer for finding this error. We have removed the bold face.

4)Line 535: The authors should compare/discuss their results on AbrB binding at PymcC to the high resolution AbrB binding map published by Chumsakul et al. (Reference 9).

We have included a comparison between the high-resolution mapping of AbrB binding sites in vivo with our in vitro analysis. Additionally, we have included a figure (**Figure 7**) that includes the position of the in vivo identified AbrB binding site within the nucleotide context of the *yvmC* promoter along with the results from our in vitro analysis.

5)Line 568-96: This is a long section with not enough relevance to warrant the lengthy treatise. Please condense.

We appreciate the comment from the reviewer. We have re-written this section for clarity and brevity.

6)Lines 648ff: The references should be carefully doublechecked. There are numerous formatting issues.

We have gone through the references and fixed the formatting issues. We thank the reviewer for pointing this out.

**References**

1.	Baier F, Gauye F, Perez-Carrasco R, Payne JL, Schaerli Y. Environment-dependent epistasis increases phenotypic diversity in gene regulatory networks. Sci Adv. 2023;9: eadf1773. doi:10.1126/sciadv.adf1773

2.	Zhu B, Stülke J. SubtiWiki in 2018: from genes and proteins to functional network annotation of the model organism *Bacillus subtilis*. Nucleic Acids Research. 2018;46: D743–D748. doi:10.1093/nar/gkx908

3.	Randazzo P, Aubert-Frambourg A, Guillot A, Auger S. The MarR-like protein PchR (YvmB) regulates expression of genes involved in pulcherriminic acid biosynthesis and in the initiation of sporulation in *Bacillus subtilis*. BMC Microbiol. 2016;16: 190. doi:10.1186/s12866-016-0807-3

4.	Chumsakul O, Takahashi H, Oshima T, Hishimoto T, Kanaya S, Ogasawara N, et al. Genome-wide binding profiles of the *Bacillus subtilis* transition state regulator AbrB and its homolog Abh reveals their interactive role in transcriptional regulation. Nucleic Acids Research. 2011;39: 414–428. doi:10.1093/nar/gkq780

5.	Chumsakul O, Nakamura K, Kurata T, Sakamoto T, Hobman JL, Ogasawara N, et al. High-Resolution Mapping of In vivo Genomic Transcription Factor Binding Sites Using In situ DNase I Footprinting and ChIP-seq. DNA Research. 2013;20: 325–338. doi:10.1093/dnares/dst013
