# Supplementary material for: Two distinct regulatory systems control pulcherrimin biosynthesis in Bacillus subtilis
Source: PLoS Genet. 2024 May 16;20(5):e1011283. doi: 10.1371/journal.pgen.1011283 (PMC11135676; doi:10.1371/journal.pgen.1011283)
Supplement: Attachment — Submitted filename: Reviewer_Response2_Fernandez.docx [file pgen.1011283.s011.docx]

Reviewer #1: The authors have addressed my concerns. Thank you for the nice paper.

That said, I want to reiterate that the definition of epistasis they are using does not conform to the classical definition of epistasis in bacterial genetics. Epistasis occurs in a double mutant when one mutation masks the phenotype of another. I think the authors definition may come from/be influenced by mis-terminology from field of evolutionary biology, a field which frequently misnames things to suit their purposes and erodes concepts I personally care about. With the evolutionary definition every double mutant can be called epistatic as something always happens when you put two mutations in the same strain, either a positive, negative or neutral effect and is therefore effectively meaningless without the pos/neg qualifier (which stands on its own), whereas the classical definition tells you the order gene products act in a particular pathway. Finally, there are already the terms additive or synergistic effects to describe such outcomes without diluting/erasing/confusing the usefulness of the term epistasis. The authors can of course do/think as they like, but as they are influential leaders in the field, I would prefer they did not reinforce a poor/misguided and ultimately uninformative redefinition. This is important to me, and I ask them to reconsider.

Dan Kearns

We appreciate the thoughtful comment from Reviewer 1. We have changed the terminology in line with the reviewer’s comment. The reviewer is correct that the use of epistasis originally used in the manuscript was influenced by the evolutionary biology field. The reason was that it provided a more quantitative assessment of gene-gene interactions while still providing information about the order of gene products in a pathway. The reviewer brings up a good point regarding the necessity of a qualifier when using the evolutionary definition, which we agree makes its use less attractive. Therefore, we have updated the manuscript and used the term “synergistic gene interactions” in lieu of epistasis to describe the effects of double and triple mutants relative to the single mutant pulcherrimin phenotypes and have reserved epistasis for instances when describing the order of gene products in the regulatory pathway.

Reviewer #2: In this manuscript, the authors provide a solid revision of their original manuscript that addresses the genetic control of the synthesis and export of the iron-chelating compound pulcherrimin in Bacillus subtilis. – In their revision of the manuscript, the authors have taken all comments/questions by the three reviewers into account and carefully addressed them in a comprehensive fashion. I have no further suggestions or questions. – Overall, this is a very nice manuscript, which significantly moves the field forward.

Reviewer #3: Thank you very much for this careful revision.

We thank reviewer 2 and 3 for their comments and appreciate their time reviewing our manuscript.
